# Supplementary material for: Content-rich biological network constructed by mining PubMed abstracts
Source: BMC Bioinformatics. 2004 Oct 8;5:147. doi: 10.1186/1471-2105-5-147 (PMC528731; doi:10.1186/1471-2105-5-147)
Supplement: Additional File 5 — The original Chilibot query results of the term "long-term potentiation (LTP)" and 22 other terms, limiting the latest references analyzed to the years 1990, 1995, 2000, and 2004. [file 1471-2105-5-147-S5.bz2 › chilibotAdditionalFile5/ltp2000/html/SYNAPTOTAGMIN.html]

 


**SYNAPTOTAGMIN** (Input: SYNAPTOTAGMIN ) 

---


**Google Searches:** Entire Web  | EDU domain only  | PDF files only

.

**External Links:** OMIM |
LocusLink
| Swissprot
| GeneCards

  
**Maps of SYNAPTOTAGMIN**

Simple
Complete
graph in
radiant
tree
square
layout.

**New Hypothesis !**

**Synonyms** 

- synaptotagmin   [PubMed]

**Synopsis**

- The results suggest the possibility that the phosphorylation of **synaptotagmin** I by CaMKII and PKC contributes to the mechanism s by which these two kinases regulate neurotransmitter release.  J Neurochem, 1999 
  [23]

- synapticvesicle proteins, e.g. **synaptotagmin**, a synaptic vesicle protein which is found in the CSF, as markers for synaptic activity or degeneration.  J Neural Transm Suppl, 1998 
  [19]

- Some muscles show very abnormal neuromuscular junctions, and some motor axon terminals are transformed into growth cone like structures embedded in **synaptotagmin** enriched vesicles.  Mol Cell Neurosci, 1999 
  [19]

- **Synaptotagmin** was found to be reduced in patients with early onset Alzheimer s disease.  J Neural Transm, 1998 
  [18]

- Here we review the role of the synaptic protein interaction synprint sites in the intracellular loop II III L II III of both alpha1B and alpha1A subunits of N type and P Q type calcium channels, which bind to syntaxin, SNAP 25, and **synaptotagmin**.  J Bioenerg Biomembr, 1998 
  [16]

- The similarity between the structures of the **synaptotagmin** I C2A domain and the PLC delta1 C2 domain suggests that the latter binds four calcium ions and acts by a similar mechanism.  Biochemistry, 1998 
  [16]

- neuromolecular studies showed increased phosphorylation of neuromodulin, increased expression of tissue type plasminogen activator, **synaptotagmin** IV and arc, which are assumed to play roles in neural outgrowth and synaptogenesis.  Nihon Shinkei Seishin Yakurigaku Zasshi, 1999 
  [16]

- Thus, N copine may have a role as a calcium sensor in postsynaptic events, in contrast to the known roles of double C2 domain containing proteins, including **synaptotagmin** I, in presynaptic events.  J Neurochem, 1999 
  [16]

- Despite the nearly constant rates of synthesis of **synaptotagmin** I, vamp 2, and synapsin I, we show that the number of SVs in these developing neurons increases,.  J Neurosci, 1997 
  [16]

- Neuroscience, 1994 
  [10]

- The eight stranded, Greek key beta sandwich fold of PKCbeta C2 is similar to that of the **synaptotagmin** I type I C2 domain.  Structure, 1998 
  [10]

- Phosphorylation dephosphorylation cycle of **synaptotagmin** can be important for regulation of recyclization of synaptic vesicles.  Biokhimiia, 1996 
  [10]

- It is likely that several different Ca binding proteins are involved in regulated exocytosis, with **synaptotagmin** apparently essential for fast exocytosis at synapses.  Essays Biochem, 1995 
  [10]

- They carry the calcium sensor **synaptotagmin**, small GTP binding proteins of the rab3 group or the v SNARE synaptobrevin.  Neurochem Int, 1997 
  [10]

- The conditions used indicate that **synaptotagmin**, as synapsin I, is phosphorylated by calcium calmodulin dependent protein kinase II.  FEBS Lett, 1993 
  [10]
